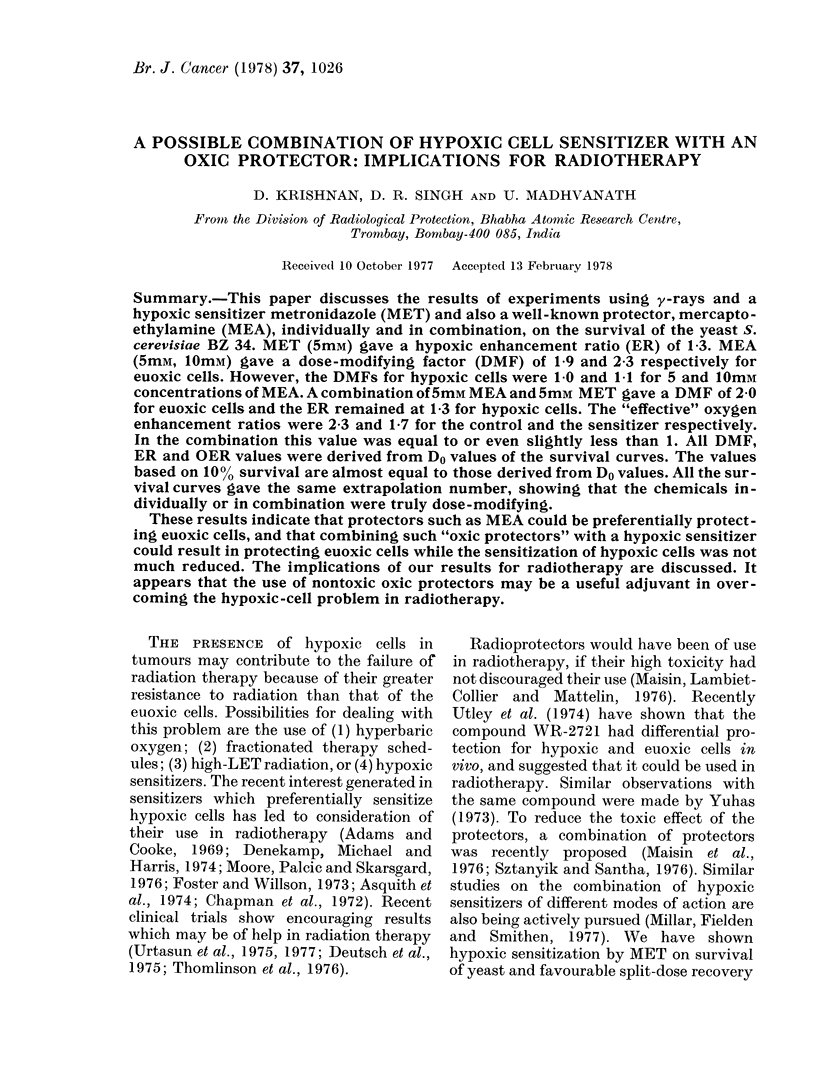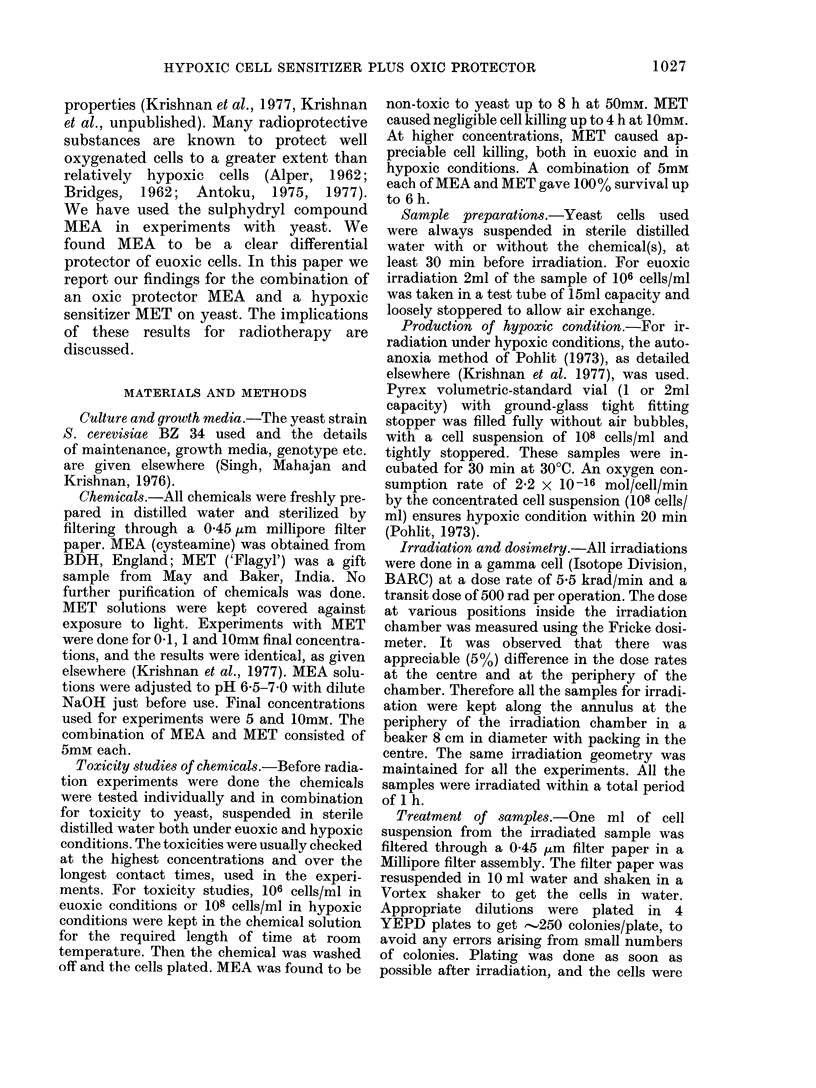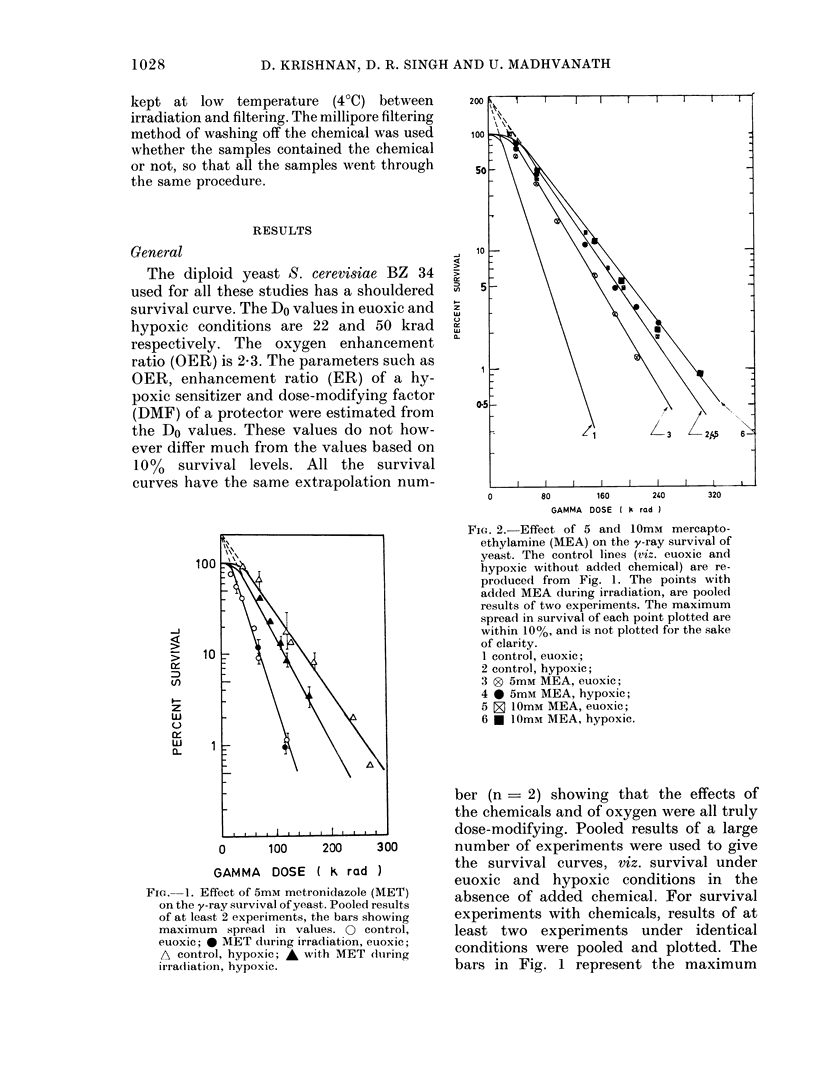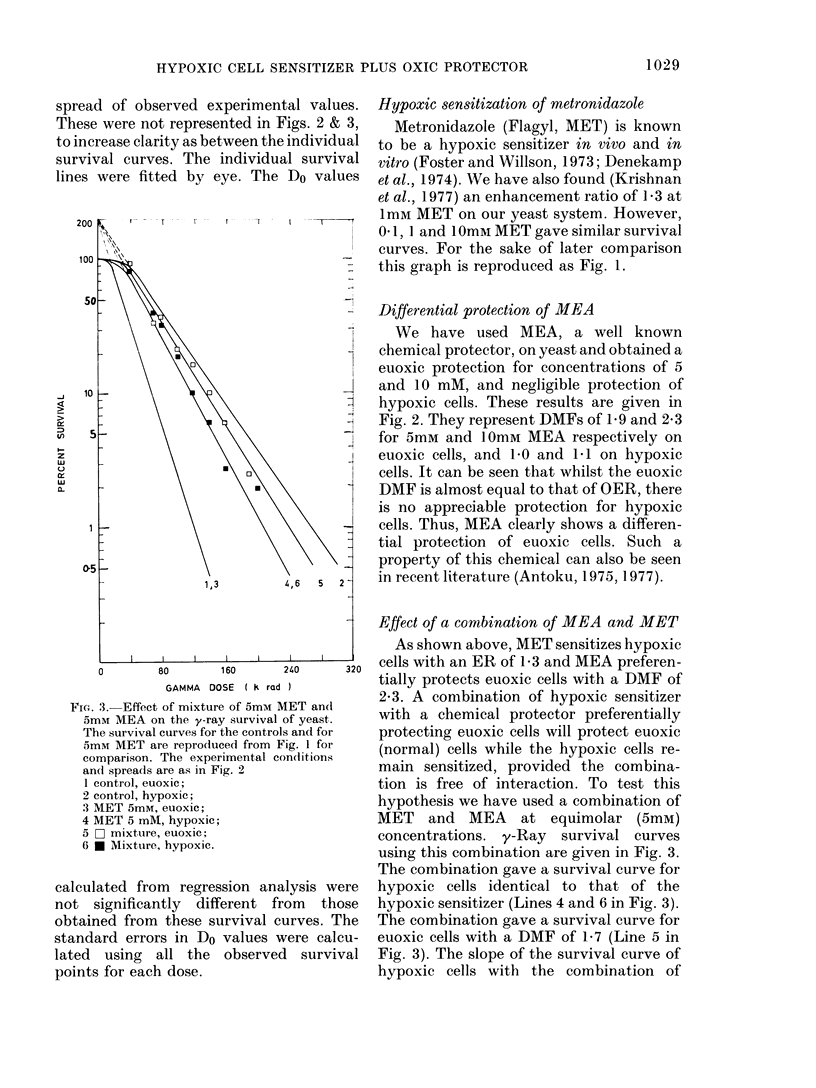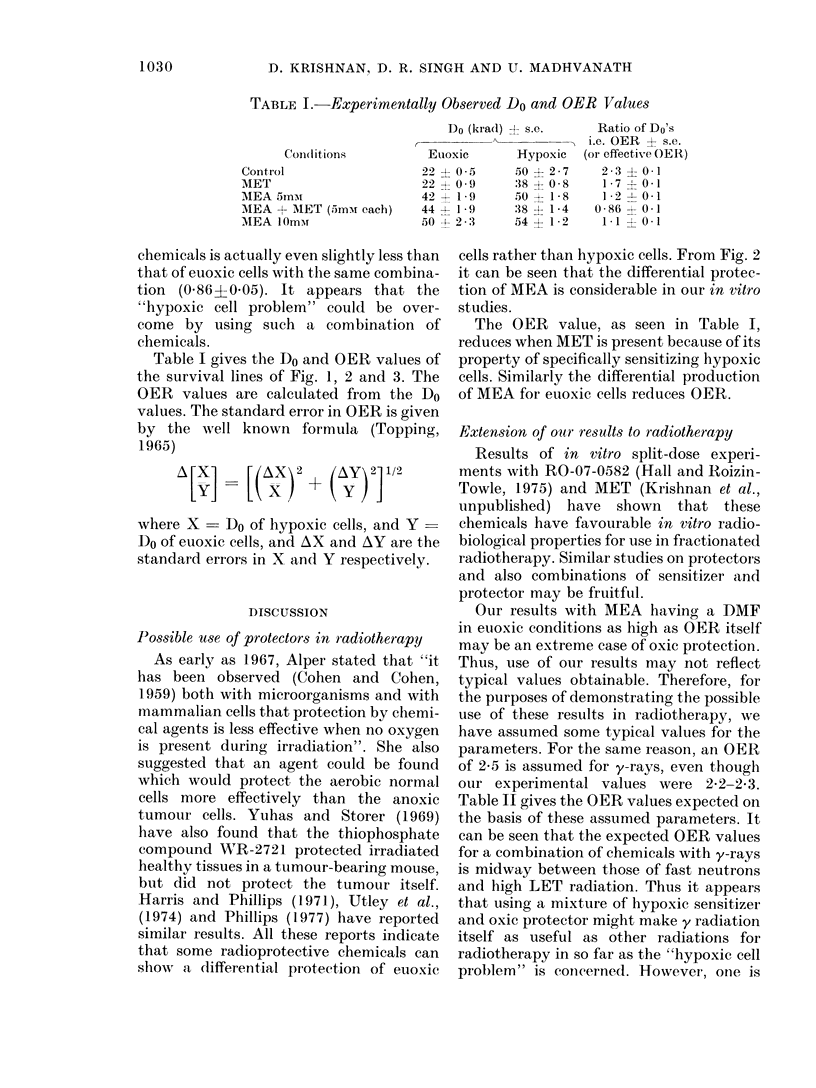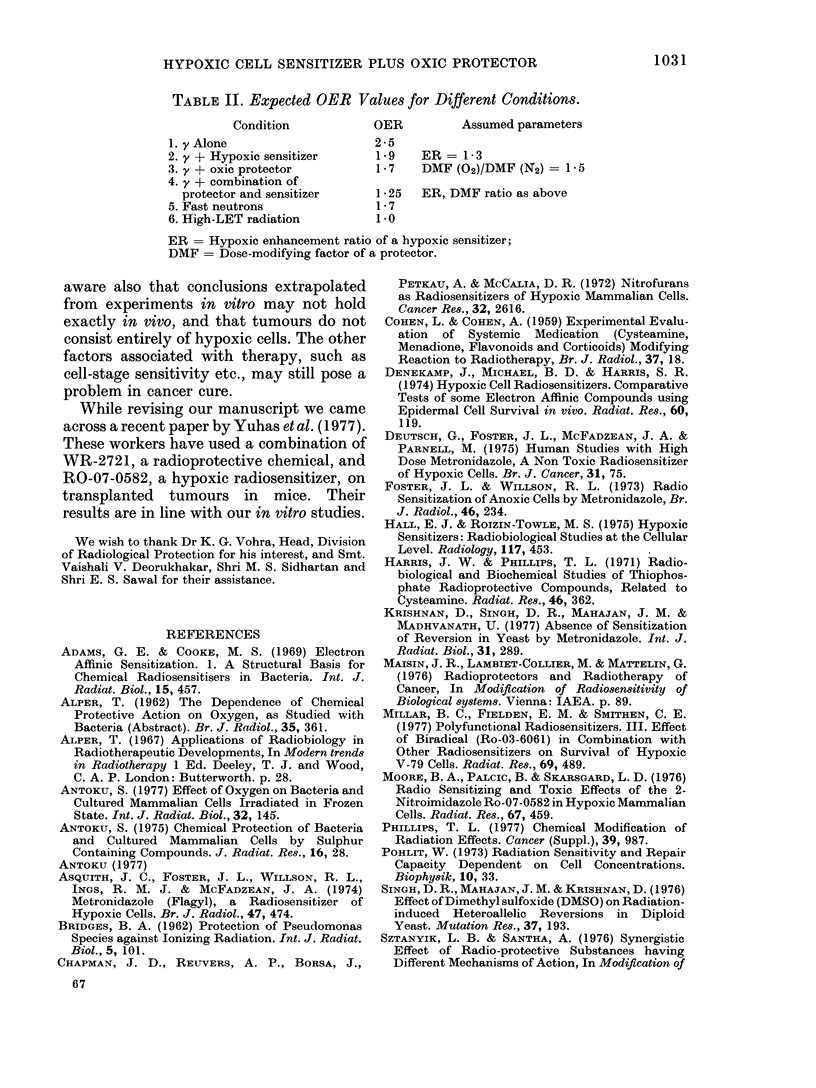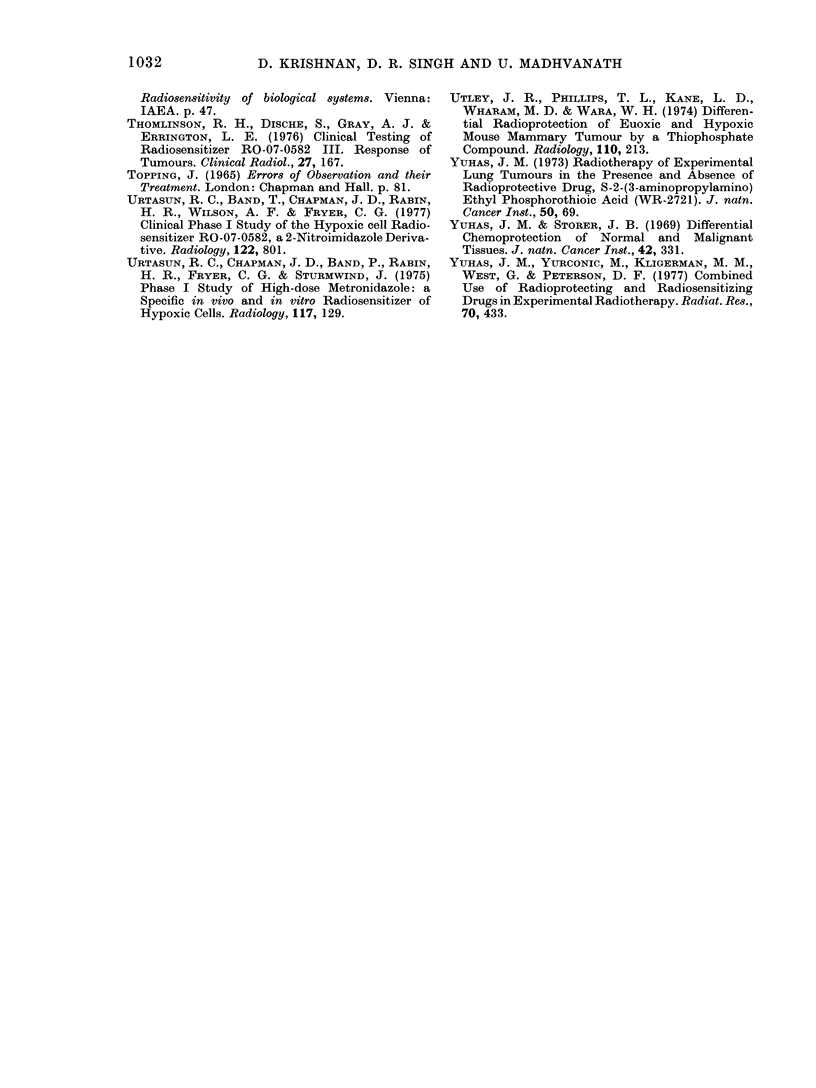# A Possible Combination of Hypoxic Cell Sensitizer with an Oxic Protector: Implications for Radiotherapy

**DOI:** 10.1038/bjc.1978.149

**Published:** 1978-06

**Authors:** D. Krishnan, D. R. Singh, U. Madhvanath

## Abstract

This paper discusses the results of experiments using γ-rays and a hypoxic sensitizer metronidazole (MET) and also a well-known protector, mercaptoethylamine (MEA), individually and in combination, on the survival of the yeast *S. cerevisiae* BZ 34. MET (5mM) gave a hypoxic enhancement ratio (ER) of 1·3. MEA (5mM, 10mM) gave a dose-modifying factor (DMF) of 1·9 and 2·3 respectively for euoxic cells. However, the DMFs for hypoxic cells were 1·0 and 1·1 for 5 and 10mM concentrations of MEA. A combination of 5mM MEA and 5mM MET gave a DMF of 2·0 for euoxic cells and the ER remained at 1·3 for hypoxic cells. The “effective” oxygen enhancement ratios were 2·3 and 1·7 for the control and the sensitizer respectively. In the combination this value was equal to or even slightly less than 1. All DMF, ER and OER values were derived from D_0_ values of the survival curves. The values based on 10% survival are almost equal to those derived from D_0_ values. All the survival curves gave the same extrapolation number, showing that the chemicals individually or in combination were truly dose-modifying.

These results indicate that protectors such as MEA could be preferentially protecting euoxic cells, and that combining such “oxic protectors” with a hypoxic sensitizer could result in protecting euoxic cells while the sensitization of hypoxic cells was not much reduced. The implications of our results for radiotherapy are discussed. It appears that the use of nontoxic oxic protectors may be a useful adjuvant in overcoming the hypoxic-cell problem in radiotherapy.


Br. J. Cancer (1978) 37, 1026

### A POSSIBLE COMBINATION OF HYPOXIC CELL SENSITIZER WITH AN OXIC PROTECTOR: IMPLICATIONS FOR RADIOTHERAPY

D. KRISHNAN, D. R. SINGH AND U. MADHVANATH

*From the Division of Radiological Protection, Bhabha Atomic Research Centre, Trombay, Bombay-400 085, India*

Received 10 October 1977 Accepted 13 February 1978

**Summary.—This paper discusses the results of experiments using $\gamma$-rays and a hypoxic sensitizer metronidazole (MET) and also a well-known protector, mercapto-ethylamine (MEA), individually and in combination, on the survival of the yeast *S. cerevisiae* BZ 34. MET (5mM) gave a hypoxic enhancement ratio (ER) of 1·3. MEA (5mM, 10mM) gave a dose-modifying factor (DMF) of 1·9 and 2·3 respectively for euoxic cells. However, the DMFs for hypoxic cells were 1·0 and 1·1 for 5 and 10mM concentrations of MEA. A combination of 5mM MEA and 5mM MET gave a DMF of 2·0 for euoxic cells and the ER remained at 1·3 for hypoxic cells. The "effective" oxygen enhancement ratios were 2·3 and 1·7 for the control and the sensitizer respectively. In the combination this value was equal to or even slightly less than 1. All DMF, ER and OER values were derived from $D_0$ values of the survival curves. The values based on 10% survival are almost equal to those derived from $D_0$ values. All the survival curves gave the same extrapolation number, showing that the chemicals individually or in combination were truly dose-modifying.**

**These results indicate that protectors such as MEA could be preferentially protecting euoxic cells, and that combining such "oxic protectors" with a hypoxic sensitizer could result in protecting euoxic cells while the sensitization of hypoxic cells was not much reduced. The implications of our results for radiotherapy are discussed. It appears that the use of nontoxic oxic protectors may be a useful adjuvant in overcoming the hypoxic-cell problem in radiotherapy.**

THE PRESENCE of hypoxic cells in tumours may contribute to the failure of radiation therapy because of their greater resistance to radiation than that of the euoxic cells. Possibilities for dealing with this problem are the use of (1) hyperbaric oxygen; (2) fractionated therapy schedules; (3) high-LET radiation, or (4) hypoxic sensitizers. The recent interest generated in sensitizers which preferentially sensitize hypoxic cells has led to consideration of their use in radiotherapy (Adams and Cooke, 1969; Denekamp, Michael and Harris, 1974; Moore, Palcic and Skarsgard, 1976; Foster and Willson, 1973; Asquith *et al.*, 1974; Chapman *et al.*, 1972). Recent clinical trials show encouraging results which may be of help in radiation therapy (Urtasun *et al.*, 1975, 1977; Deutsch *et al.*, 1975; Thomlinson *et al.*, 1976).

Radioprotectors would have been of use in radiotherapy, if their high toxicity had not discouraged their use (Maisin, Lambiet-Collier and Mattelin, 1976). Recently Utley *et al.* (1974) have shown that the compound WR-2721 had differential protection for hypoxic and euoxic cells *in vivo*, and suggested that it could be used in radiotherapy. Similar observations with the same compound were made by Yuhas (1973). To reduce the toxic effect of the protectors, a combination of protectors was recently proposed (Maisin *et al.*, 1976; Sztanyik and Santha, 1976). Similar studies on the combination of hypoxic sensitizers of different modes of action are also being actively pursued (Millar, Fielden and Smithen, 1977). We have shown hypoxic sensitization by MET on survival of yeast and favourable split-dose recovery properties (Krishnan *et al.*, 1977, Krishnan *et al.*, unpublished). Many radioprotective substances are known to protect well oxygenated cells to a greater extent than relatively hypoxic cells (Alper, 1962; Bridges, 1962; Antoku, 1975, 1977). We have used the sulphydryl compound MEA in experiments with yeast. We found MEA to be a clear differential protector of euoxic cells. In this paper we report our findings for the combination of an oxic protector MEA and a hypoxic sensitizer MET on yeast. The implications of these results for radiotherapy are discussed.

### MATERIALS AND METHODS

*Culture and growth media.*—The yeast strain *S. cerevisiae* BZ 34 used and the details of maintenance, growth media, genotype etc. are given elsewhere (Singh, Mahajan and Krishnan, 1976).

*Chemicals.*—All chemicals were freshly prepared in distilled water and sterilized by filtering through a 0·45 $\mu$m millipore filter paper. MEA (cysteamine) was obtained from BDH, England; MET ('Flagyl') was a gift sample from May and Baker, India. No further purification of chemicals was done. MET solutions were kept covered against exposure to light. Experiments with MET were done for 0·1, 1 and 10mM final concentrations, and the results were identical, as given elsewhere (Krishnan *et al.*, 1977). MEA solutions were adjusted to pH 6·5–7·0 with dilute NaOH just before use. Final concentrations used for experiments were 5 and 10mM. The combination of MEA and MET consisted of 5mM each.

*Toxicity studies of chemicals.*—Before radiation experiments were done the chemicals were tested individually and in combination for toxicity to yeast, suspended in sterile distilled water both under euoxic and hypoxic conditions. The toxicities were usually checked at the highest concentrations and over the longest contact times, used in the experiments. For toxicity studies, $10^6$ cells/ml in euoxic conditions or $10^8$ cells/ml in hypoxic conditions were kept in the chemical solution for the required length of time at room temperature. Then the chemical was washed off and the cells plated. MEA was found to be non-toxic to yeast up to 8 h at 50mM. MET caused negligible cell killing up to 4 h at 10mM. At higher concentrations, MET caused appreciable cell killing, both in euoxic and in hypoxic conditions. A combination of 5mM each of MEA and MET gave 100% survival up to 6 h.

*Sample preparations.*—Yeast cells used were always suspended in sterile distilled water with or without the chemical(s), at least 30 min before irradiation. For euoxic irradiation 2ml of the sample of $10^6$ cells/ml was taken in a test tube of 15ml capacity and loosely stoppered to allow air exchange.

*Production of hypoxic condition.*—For irradiation under hypoxic conditions, the auto-anoxia method of Pohlit (1973), as detailed elsewhere (Krishnan *et al.* 1977), was used. Pyrex volumetric-standard vial (1 or 2ml capacity) with ground-glass tight fitting stopper was filled fully without air bubbles, with a cell suspension of $10^8$ cells/ml and tightly stoppered. These samples were incubated for 30 min at 30°C. An oxygen consumption rate of $2{\cdot}2 \times 10^{-16}$ mol/cell/min by the concentrated cell suspension ($10^8$ cells/ml) ensures hypoxic condition within 20 min (Pohlit, 1973).

*Irradiation and dosimetry.*—All irradiations were done in a gamma cell (Isotope Division, BARC) at a dose rate of 5·5 krad/min and a transit dose of 500 rad per operation. The dose at various positions inside the irradiation chamber was measured using the Fricke dosimeter. It was observed that there was appreciable (5%) difference in the dose rates at the centre and at the periphery of the chamber. Therefore all the samples for irradiation were kept along the annulus at the periphery of the irradiation chamber in a beaker 8 cm in diameter with packing in the centre. The same irradiation geometry was maintained for all the experiments. All the samples were irradiated within a total period of 1 h.

*Treatment of samples.*—One ml of cell suspension from the irradiated sample was filtered through a 0·45 $\mu$m filter paper in a Millipore filter assembly. The filter paper was resuspended in 10 ml water and shaken in a Vortex shaker to get the cells in water. Appropriate dilutions were plated in 4 YEPD plates to get ~250 colonies/plate, to avoid any errors arising from small numbers of colonies. Plating was done as soon as possible after irradiation, and the cells were kept at low temperature (4°C) between irradiation and filtering. The millipore filtering method of washing off the chemical was used whether the samples contained the chemical or not, so that all the samples went through the same procedure.

### RESULTS

### *General*

The diploid yeast *S. cerevisiae* BZ 34 used for all these studies has a shouldered survival curve. The $D_0$ values in euoxic and hypoxic conditions are 22 and 50 krad respectively. The oxygen enhancement ratio (OER) is 2·3. The parameters such as OER, enhancement ratio (ER) of a hypoxic sensitizer and dose-modifying factor (DMF) of a protector were estimated from the $D_0$ values. These values do not however differ much from the values based on 10% survival levels. All the survival curves have the same extrapolation num-

FIG.—1. Effect of 5mM metronidazole (MET) on the γ-ray survival of yeast. Pooled results of at least 2 experiments, the bars showing maximum spread in values. ○ control, euoxic; ● MET during irradiation, euoxic; △ control, hypoxic; ▲ with MET during irradiation, hypoxic.

FIG. 2.—Effect of 5 and 10mM mercapto-ethylamine (MEA) on the γ-ray survival of yeast. The control lines (*viz.* euoxic and hypoxic without added chemical) are reproduced from Fig. 1. The points with added MEA during irradiation, are pooled results of two experiments. The maximum spread in survival of each point plotted are within 10%, and is not plotted for the sake of clarity.
1 control, euoxic;
2 control, hypoxic;
3 ⊗ 5mM MEA, euoxic;
4 ● 5mM MEA, hypoxic;
5 ⊠ 10mM MEA, euoxic;
6 ■ 10mM MEA, hypoxic.

ber (n = 2) showing that the effects of the chemicals and of oxygen were all truly dose-modifying. Pooled results of a large number of experiments were used to give the survival curves, *viz.* survival under euoxic and hypoxic conditions in the absence of added chemical. For survival experiments with chemicals, results of at least two experiments under identical conditions were pooled and plotted. The bars in Fig. 1 represent the maximum spread of observed experimental values. These were not represented in Figs. 2 & 3, to increase clarity as between the individual survival curves. The individual survival lines were fitted by eye. The $D_0$ values

FIG. 3.—Effect of mixture of 5mM MET and 5mM MEA on the γ-ray survival of yeast. The survival curves for the controls and for 5mM MET are reproduced from Fig. 1 for comparison. The experimental conditions and spreads are as in Fig. 2
1 control, euoxic;
2 control, hypoxic;
3 MET 5mM, euoxic;
4 MET 5 mM, hypoxic;
5 □ mixture, euoxic;
6 ■ Mixture, hypoxic.

calculated from regression analysis were not significantly different from those obtained from these survival curves. The standard errors in $D_0$ values were calculated using all the observed survival points for each dose.

*Hypoxic sensitization of metronidazole*

Metronidazole (Flagyl, MET) is known to be a hypoxic sensitizer *in vivo* and *in vitro* (Foster and Willson, 1973; Denekamp *et al.*, 1974). We have also found (Krishnan *et al.*, 1977) an enhancement ratio of 1·3 at 1mM MET on our yeast system. However, 0·1, 1 and 10mM MET gave similar survival curves. For the sake of later comparison this graph is reproduced as Fig. 1.

*Differential protection of MEA*

We have used MEA, a well known chemical protector, on yeast and obtained a euoxic protection for concentrations of 5 and 10 mM, and negligible protection of hypoxic cells. These results are given in Fig. 2. They represent DMFs of 1·9 and 2·3 for 5mM and 10mM MEA respectively on euoxic cells, and 1·0 and 1·1 on hypoxic cells. It can be seen that whilst the euoxic DMF is almost equal to that of OER, there is no appreciable protection for hypoxic cells. Thus, MEA clearly shows a differential protection of euoxic cells. Such a property of this chemical can also be seen in recent literature (Antoku, 1975, 1977).

*Effect of a combination of MEA and MET*

As shown above, MET sensitizes hypoxic cells with an ER of 1·3 and MEA preferentially protects euoxic cells with a DMF of 2·3. A combination of hypoxic sensitizer with a chemical protector preferentially protecting euoxic cells will protect euoxic (normal) cells while the hypoxic cells remain sensitized, provided the combination is free of interaction. To test this hypothesis we have used a combination of MET and MEA at equimolar (5mM) concentrations. γ-Ray survival curves using this combination are given in Fig. 3. The combination gave a survival curve for hypoxic cells identical to that of the hypoxic sensitizer (Lines 4 and 6 in Fig. 3). The combination gave a survival curve for euoxic cells with a DMF of 1·7 (Line 5 in Fig. 3). The slope of the survival curve of hypoxic cells with the combination of

TABLE I.—*Experimentally Observed $D_0$ and OER Values*

| Conditions | $D_0$ (krad) ± s.e. Euoxic | Hypoxic | Ratio of $D_0$'s i.e. OER ± s.e. (or effective OER) |
|---|---|---|---|
| Control | 22 ± 0·5 | 50 ± 2·7 | 2·3 ± 0·1 |
| MET | 22 ± 0·9 | 38 ± 0·8 | 1·7 ± 0·1 |
| MEA 5mM | 42 ± 1·9 | 50 ± 1·8 | 1·2 ± 0·1 |
| MEA + MET (5mM each) | 44 ± 1·9 | 38 ± 1·4 | 0·86 ± 0·1 |
| MEA 10mM | 50 ± 2·3 | 54 ± 1·2 | 1·1 ± 0·1 |

chemicals is actually even slightly less than that of euoxic cells with the same combination (0·86±0·05). It appears that the "hypoxic cell problem" could be overcome by using such a combination of chemicals.

Table I gives the $D_0$ and OER values of the survival lines of Fig. 1, 2 and 3. The OER values are calculated from the $D_0$ values. The standard error in OER is given by the well known formula (Topping, 1965)

$$\Delta\left[\frac{X}{Y}\right] = \left[\left(\frac{\Delta X}{X}\right)^2 + \left(\frac{\Delta Y}{Y}\right)^2\right]^{1/2}$$

where X = $D_0$ of hypoxic cells, and Y = $D_0$ of euoxic cells, and $\Delta X$ and $\Delta Y$ are the standard errors in X and Y respectively.

### DISCUSSION

### *Possible use of protectors in radiotherapy*

As early as 1967, Alper stated that "it has been observed (Cohen and Cohen, 1959) both with microorganisms and with mammalian cells that protection by chemical agents is less effective when no oxygen is present during irradiation". She also suggested that an agent could be found which would protect the aerobic normal cells more effectively than the anoxic tumour cells. Yuhas and Storer (1969) have also found that the thiophosphate compound WR-2721 protected irradiated healthy tissues in a tumour-bearing mouse, but did not protect the tumour itself. Harris and Phillips (1971), Utley *et al.*, (1974) and Phillips (1977) have reported similar results. All these reports indicate that some radioprotective chemicals can show a differential protection of euoxic cells rather than hypoxic cells. From Fig. 2 it can be seen that the differential protection of MEA is considerable in our *in vitro* studies.

The OER value, as seen in Table I, reduces when MET is present because of its property of specifically sensitizing hypoxic cells. Similarly the differential production of MEA for euoxic cells reduces OER.

### *Extension of our results to radiotherapy*

Results of *in vitro* split-dose experiments with RO-07-0582 (Hall and Roizin-Towle, 1975) and MET (Krishnan *et al.*, unpublished) have shown that these chemicals have favourable *in vitro* radiobiological properties for use in fractionated radiotherapy. Similar studies on protectors and also combinations of sensitizer and protector may be fruitful.

Our results with MEA having a DMF in euoxic conditions as high as OER itself may be an extreme case of oxic protection. Thus, use of our results may not reflect typical values obtainable. Therefore, for the purposes of demonstrating the possible use of these results in radiotherapy, we have assumed some typical values for the parameters. For the same reason, an OER of 2·5 is assumed for $\gamma$-rays, even though our experimental values were 2·2–2·3. Table II gives the OER values expected on the basis of these assumed parameters. It can be seen that the expected OER values for a combination of chemicals with $\gamma$-rays is midway between those of fast neutrons and high LET radiation. Thus it appears that using a mixture of hypoxic sensitizer and oxic protector might make $\gamma$ radiation itself as useful as other radiations for radiotherapy in so far as the "hypoxic cell problem" is concerned. However, one is

TABLE II. *Expected OER Values for Different Conditions.*

| Condition | OER | Assumed parameters |
|---|---|---|
| 1. $\gamma$ Alone | 2·5 | |
| 2. $\gamma$ + Hypoxic sensitizer | 1·9 | ER = 1·3 |
| 3. $\gamma$ + oxic protector | 1·7 | DMF ($O_2$)/DMF ($N_2$) = 1·5 |
| 4. $\gamma$ + combination of protector and sensitizer | 1·25 | ER, DMF ratio as above |
| 5. Fast neutrons | 1·7 | |
| 6. High-LET radiation | 1·0 | |

ER = Hypoxic enhancement ratio of a hypoxic sensitizer;
DMF = Dose-modifying factor of a protector.

aware also that conclusions extrapolated from experiments *in vitro* may not hold exactly *in vivo*, and that tumours do not consist entirely of hypoxic cells. The other factors associated with therapy, such as cell-stage sensitivity etc., may still pose a problem in cancer cure.

While revising our manuscript we came across a recent paper by Yuhas *et al.* (1977). These workers have used a combination of WR-2721, a radioprotective chemical, and RO-07-0582, a hypoxic radiosensitizer, on transplanted tumours in mice. Their results are in line with our *in vitro* studies.

We wish to thank Dr K. G. Vohra, Head, Division of Radiological Protection for his interest, and Smt. Vaishali V. Deorukhakar, Shri M. S. Sidhartan and Shri E. S. Sawal for their assistance.

### REFERENCES

ADAMS, G. E. & COOKE, M. S. (1969) Electron Affinic Sensitization. 1. A Structural Basis for Chemical Radiosensitisers in Bacteria. *Int. J. Radiat. Biol.*, **15**, 457.

ALPER, T. (1962) The Dependence of Chemical Protective Action on Oxygen, as Studied with Bacteria (Abstract). *Br. J. Radiol.*, **35**, 361.

ALPER, T. (1967) Applications of Radiobiology in Radiotherapeutic Developments, In *Modern trends in Radiotherapy* 1 Ed. Deeley, T. J. and Wood, C. A. P. London: Butterworth. p. 28.

ANTOKU, S. (1977) Effect of Oxygen on Bacteria and Cultured Mammalian Cells Irradiated in Frozen State. *Int. J. Radiat. Biol.*, **32**, 145.

ANTOKU, S. (1975) Chemical Protection of Bacteria and Cultured Mammalian Cells by Sulphur Containing Compounds. *J. Radiat. Res.*, **16**, 28.

ANTOKU (1977)

ASQUITH, J. C., FOSTER, J. L., WILLSON, R. L., INGS, R. M. J. & MCFADZEAN, J. A. (1974) Metronidazole (Flagyl), a Radiosensitizer of Hypoxic Cells. *Br. J. Radiol.*, **47**, 474.

BRIDGES, B. A. (1962) Protection of Pseudomonas Species against Ionizing Radiation. *Int. J. Radiat. Biol.*, **5**, 101.

CHAPMAN, J. D., REUVERS, A. P., BORSA, J., PETKAU, A. & MCCALIA, D. R. (1972) Nitrofurans as Radiosensitizers of Hypoxic Mammalian Cells. *Cancer Res.*, **32**, 2616.

COHEN, L. & COHEN, A. (1959) Experimental Evaluation of Systemic Medication (Cysteamine, Menadione, Flavonoids and Corticoids) Modifying Reaction to Radiotherapy, *Br. J. Radiol.*, **37**, 18.

DENEKAMP, J., MICHAEL, B. D. & HARRIS, S. R. (1974) Hypoxic Cell Radiosensitizers. Comparative Tests of some Electron Affinic Compounds using Epidermal Cell Survival *in vivo*. *Radiat. Res.*, **60**, 119.

DEUTSCH, G., FOSTER, J. L., MCFADZEAN, J. A. & PARNELL, M. (1975) Human Studies with High Dose Metronidazole, A Non Toxic Radiosensitizer of Hypoxic Cells. *Br. J. Cancer*, **31**, 75.

FOSTER, J. L. & WILLSON, R. L. (1973) Radio Sensitization of Anoxic Cells by Metronidazole, *Br. J. Radiol.*, **46**, 234.

HALL, E. J. & ROIZIN-TOWLE, M. S. (1975) Hypoxic Sensitizers: Radiobiological Studies at the Cellular Level. *Radiology*, **117**, 453.

HARRIS, J. W. & PHILLIPS, T. L. (1971) Radiobiological and Biochemical Studies of Thiophosphate Radioprotective Compounds, Related to Cysteamine. *Radiat. Res.*, **46**, 362.

KRISHNAN, D., SINGH, D. R., MAHAJAN, J. M. & MADHVANATH, U. (1977) Absence of Sensitization of Reversion in Yeast by Metronidazole. *Int. J. Radiat. Biol.*, **31**, 289.

MAISIN, J. R., LAMBIET-COLLIER, M. & MATTELIN, G. (1976) Radioprotectors and Radiotherapy of Cancer, In *Modification of Radiosensitivity of Biological systems*. Vienna: IAEA. p. 89.

MILLAR, B. C., FIELDEN, E. M. & SMITHEN, C. E. (1977) Polyfunctional Radiosensitizers. III. Effect of Biradical (Ro-03-6061) in Combination with Other Radiosensitizers on Survival of Hypoxic V-79 Cells. *Radiat. Res.*, **69**, 489.

MOORE, B. A., PALCIC, B. & SKARSGARD, L. D. (1976) Radio Sensitizing and Toxic Effects of the 2-Nitroimidazole Ro-07-0582 in Hypoxic Mammalian Cells. *Radiat. Res.*, **67**, 459.

PHILLIPS, T. L. (1977) Chemical Modification of Radiation Effects. *Cancer* (Suppl.), **39**, 987.

POHLIT, W. (1973) Radiation Sensitivity and Repair Capacity Dependent on Cell Concentrations. *Biophysik*, **10**, 33.

SINGH, D. R., MAHAJAN, J. M. & KRISHNAN, D. (1976) Effect of Dimethyl sulfoxide (DMSO) on Radiation-induced Heteroallelic Reversions in Diploid Yeast. *Mutation Res.*, **37**, 193.

SZTANYIK, L. B. & SANTHA, A. (1976) Synergistic Effect of Radio-protective Substances having Different Mechanisms of Action, In *Modification of*

*Radiosensitivity of biological systems.* Vienna: IAEA. p. 47.

THOMLINSON, R. H., DISCHE, S., GRAY, A. J. & ERRINGTON, L. E. (1976) Clinical Testing of Radiosensitizer RO-07-0582 III. Response of Tumours. *Clinical Radiol.*, **27**, 167.

TOPPING, J. (1965) *Errors of Observation and their Treatment.* London: Chapman and Hall. p. 81.

URTASUN, R. C., BAND, T., CHAPMAN, J. D., RABIN, H. R., WILSON, A. F. & FRYER, C. G. (1977) Clinical Phase I Study of the Hypoxic cell Radiosensitizer RO-07-0582, a 2-Nitroimidazole Derivative. *Radiology*, **122**, 801.

URTASUN, R. C., CHAPMAN, J. D., BAND, P., RABIN, H. R., FRYER, C. G. & STURMWIND, J. (1975) Phase I Study of High-dose Metronidazole: a Specific *in vivo* and *in vitro* Radiosensitizer of Hypoxic Cells. *Radiology*, **117**, 129.

UTLEY, J. R., PHILLIPS, T. L., KANE, L. D., WHARAM, M. D. & WARA, W. H. (1974) Differential Radioprotection of Euoxic and Hypoxic Mouse Mammary Tumour by a Thiophosphate Compound. *Radiology*, **110**, 213.

YUHAS, J. M. (1973) Radiotherapy of Experimental Lung Tumours in the Presence and Absence of Radioprotective Drug, S-2-(3-aminopropylamino) Ethyl Phosphorothioic Acid (WR-2721). *J. natn. Cancer Inst.*, **50**, 69.

YUHAS, J. M. & STORER, J. B. (1969) Differential Chemoprotection of Normal and Malignant Tissues. *J. natn. Cancer Inst.*, **42**, 331.

YUHAS, J. M., YURCONIC, M., KLIGERMAN, M. M., WEST, G. & PETERSON, D. F. (1977) Combined Use of Radioprotecting and Radiosensitizing Drugs in Experimental Radiotherapy. *Radiat. Res.*, **70**, 433.